# A Case of Acromegaly With Progressed Diabetic Retinopathy and Sarcopenia Diagnosed Following the Onset of Severe Hypoglycemia

**DOI:** 10.7759/cureus.58461

**Published:** 2024-04-17

**Authors:** Haremaru Kubo, Kazuhiro Sugimoto, Ryota Wada, Naohiro Sekikawa, Minoru Inoue

**Affiliations:** 1 Diabetes Center, Ohta Nishinouchi Hospital, Koriyama, JPN; 2 Diabetes, Endocrinology, and Metabolism, Kitasato University School of Medicine, Sagamihara, JPN; 3 Internal Medicine, Ohta Nishinouchi Hospital, Koriyama, JPN

**Keywords:** diabetic retinopathy, hypoglycemia, insulin autoimmune disease, sarcopenia, acromegaly

## Abstract

Acromegaly is a rare disorder characterized by excessive production of growth hormone (GH) from a pituitary tumor, typically leading to elevated glucose levels due to increased insulin resistance; hypoglycemia is rare. However, the long-term effect of excess GH on the peripheral organs is still unclear. Here we present a 69-year-old man evaluated for the cause of a hypoglycemic episode. He was underweight (body mass index: 17.3 kg/m^2^) with sarcopenia, which potentially contributed to his hypoglycemia. Notably, he exhibited progressed proliferative diabetic retinopathy compared to other microvascular complications, leading to further endocrinological investigation. As a result, he was diagnosed with acromegaly showing elevated GH and insulin-like growth factor-1 (IGF-1) with a pituitary tumor. Opting against transsphenoidal surgery (TSS), the patient was treated with a somatostatin analog (SSA), achieving normalized IGF-1 levels with a monthly 120 mg lanreotide injection. In this case, acromegaly could lead to sarcopenia from GH-derived gluconeogenesis in the peripheral organs such as the reduction of muscle leading to reduced glucose reserves. Acromegaly in the elderly may present atypicality. Clinicians should be vigilant for unique manifestations such as advanced diabetic retinopathy, even in elderly patients with hypoglycemia.

## Introduction

Acromegaly is a rare disease that shows many complications derived from excess growth hormone (GH) from pituitary tumors [1]. To decrease the risk of complications from acromegaly-related comorbidities such as cardiac events, the treatment goal in acromegaly is normalized insulin-like growth factor-1 (IGF-1) levels [1,2]. The first choice of treatment in acromegaly is surgical resection of GH-secreting pituitary neuroendocrine tumor via transsphenoidal surgery (TSS). However, some patients are treated with somatostatin analogs (SSA) because of surgical intolerance or their decision, especially in elderly patients [3]. Among acromegaly-related complications, diabetes is the most frequent metabolic comorbidity presented in 30-50% of acromegaly patients [4]. The mechanism of glucose intolerance in acromegaly is thought as (1) increased insulin resistance via increased free fatty acids via GH-induced lipolysis with the theory of post-receptor inhibition of insulin signaling and (2) increased gluconeogenesis in the liver and kidney controlling key gluconeogenic enzymes, including phosphoenolpyruvate carboxykinase 1 and glucose-6 phosphatase [5-7]. Therefore, including secondary diabetes, pre-existing diabetes also could be worsened by acromegaly. Interestingly, among diabetes-related microangiopathies, diabetic retinopathy is significantly progressed in patients with acromegaly through GH-signaling, which could be the clue for the diagnosis of acromegaly [8,9].

Hypoglycemia is sometimes shown in patients with diabetes, and one of the substantial factors progressing diabetic retinopathy via local hypoxia in the retina [10,11]. Hypoglycemia is induced by several causes, such as oral hypoglycemic agents (OHAs), insulin injections, and antibodies to insulin and insulin-secreting tumors [12]. Otherwise, because of the elevated insulin resistance written above, usually, hypoglycemia could not happen in patients with acromegaly. Therefore, the patients who show hypoglycemia are not typically suspected of accompanying acromegaly.

Herein, we report the case of acromegaly with severe sarcopenia in an elderly patient, which was diagnosed via the investigation of hypoglycemia.

## Case presentation

A 69-year-old man visited our hospital for the investigation of hypoglycemia. He was diagnosed with type 2 diabetes in his 40s and followed up at a clinic. Though he temporarily used insulin injection (insulin degludec), his medication was gradually changed to oral OHA, metformin 1500 mg and vildagliptin 100 mg, from age 60. Though his microvascular complication was not fully analyzed in the clinic, his renal function was maintained and proteinuria was negative within outpatient. He had no history of macrovascular complications. His left eye lost vision due to diabetic retinopathy at age 64. After that, his body weight gradually decreased from around 60 kg to 50 kg over several years along with decreased daily activities due to the blindness of his left eye. Within this period, he did not show hyperphagia, polydipsia, or polyuria, suggesting metabolic failure. Otherwise, food intake gradually decreased along with decreased activity. Before admission to the previous hospital, his medication was metformin 1500 mg, vildagliptin 100 mg, iansoprazole 15 mg, magnesium oxide 990 mg, and sennosides 12 mg. At age 68, he suddenly had a hypoglycemia attack (22 mg/dL) and was transferred to the previous hospital. OHAs were stopped, and he was treated with intravenous one-shot glucose infusion as 20mL of 50% glucose, followed by continuous glucose infusion (maltose 25000 mg/day) for several days. After that, his glucose level gradually increased and the hypoglycemia attack was not repeated. Thereafter, he was referred to our hospital.

**Figure 1:**
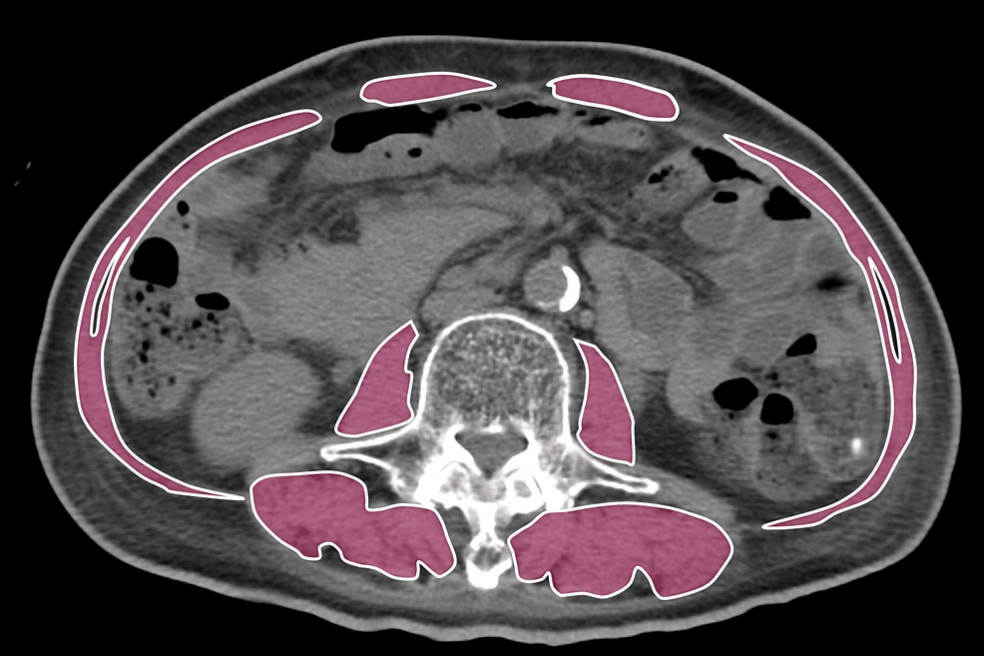
Representative abdominal CT images of sarcopenia Demonstrable axial CT images at the third lumbar vertebra region with highlighted skeletal muscles. The image was obtained from LightSpeed VCT Vision (GE Healthcare, Princeton, NJ, USA) and analyzed with Ziostation2 (Ziosoft, Tokyo, Japan). CT, computed tomography

At first, we suspected sarcopenia with malnutrition as the cause of hypoglycemia, especially when he showed severe emaciation in addition to chronic imbalanced diabetes. He did not show any specific symptoms such as palpitations or trembling suggesting hypoglycemia at first appearance. His consciousness was normal with a blood pressure of 135/70 mmHg and a pulse rate of 79 betas/min, and there was no rale, murmur, or extracardiac sound in chest auscultation. There was no other significant finding. His medication was already stopped for several days at the previous hospital. Grip strength (5.9 kg and 5.8 kg at right and left; <26 kg), walking speed (0.45 m/sec; ≤0.8 m/sec), and skeletal muscle index (SMI) at the third lumbar vertebra level in a computed tomography (CT) image-calculated area (29.4 cm^2^/m^2^; ≤42.0 cm^2^/m^2^) suggested apparent sarcopenia (Figure 1) [13,14].

**Figure 2:**
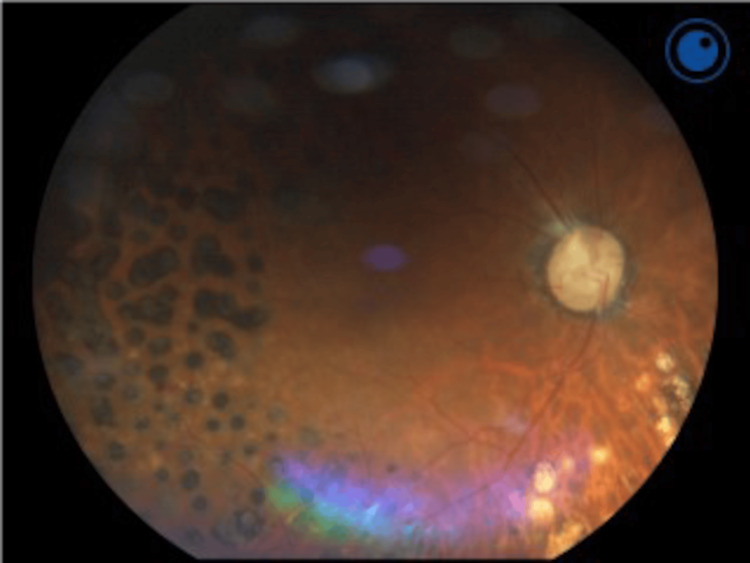
An image of diabetic retinopathy in the right eye Fundus photography of the present case was shown. Laser photocoagulation for proliferative diabetic retinopathy was repeated many times previously.

His body weight was still 50 kg (body height: 170 cm and body mass index: 17.30 kg/m^2^) at first appearance. Low serum albumin (3 g/dL), low-density lipoprotein cholesterol (61 mg/dL), and total cholesterol (110 mg/dL) level at laboratory test suggested malnutrition accompanying sarcopenia. There was no evidence of chronic liver disease and no history of gastrectomy or other causes of dumping syndrome. Otherwise, an anti-insulin antibody was positive (3.6 U/mL; reference range <0.4 U/mL), possibly affecting the hypoglycemia attack. However, we could not confirm other hypoglycemia events. Endogenous insulin secretion seemed to be relatively preserved (immunoreactive insulin and C-peptide levels were 5.1 mU/mL and 1.97 ng/mL at fasting glucose level 275 mg/dL in the first visit after intravenous glucose infusion in the previous hospital). Comprehensively, all these factors above were thought to be associated with the hypoglycemia attack. Hypoglycemia is known as the accelerator of diabetic retinopathy; we analyzed his diabetic microangiopathy and the resulting diabetic retinopathy was outstandingly progressed compared with other complications (retinopathy; proliferative diabetic retinopathy after laser photocoagulation shown in Figure 2; lost vision at the left eye; nephropathy; G1A2 and neuropathy; decreased bilateral ankle reflex; and vibration sensations without the presence of symptoms).

**Figure 3:**
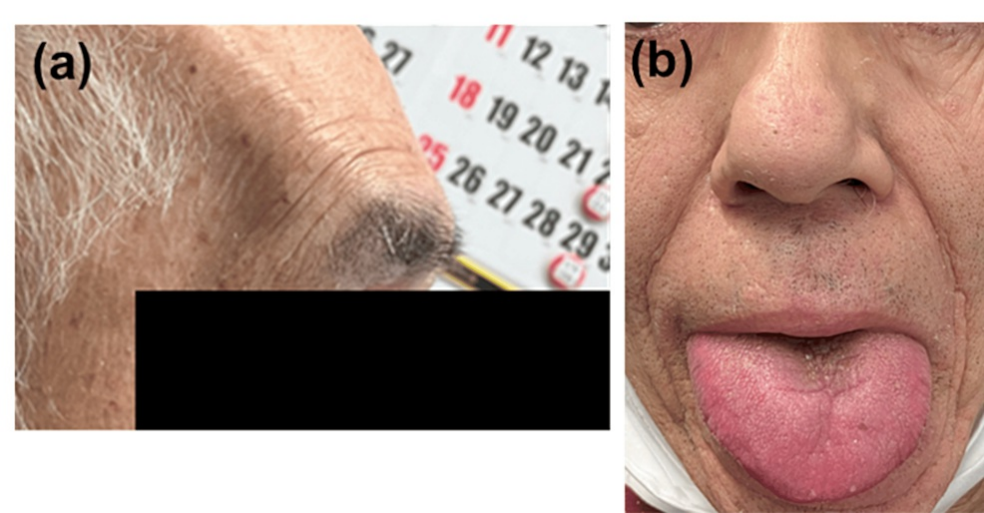
Images of acromegalic phenotypes The phenotype led to suspicion of acromegaly. The facial features were prominent brow ridges (a), macroglossia, and thickened nose (b).

There was no evidence of impaired cardiopulmonary dysfunction through transthoracic echocardiography and electrocardiogram. In the neurological examination, carpal tunnel syndrome was additionally suggested. Moreover, further physical examination revealed acromegalic features (prominent brow ridges, macroglossia, and thickened nose) (Figure 3).

Excess of GH-IGF-1 axis was confirmed with elevated GH of 4.42 ng/mL and IGF-1 of 364 ng/mL (+4.70 standard deviation (SD)) at elevated fasting plasma glucose level of 245 mg/dL at morning baseline blood sample. A 75 g oral glucose tolerance test was omitted because unsuppressed GH was already confirmed at hyperglycemic condition, and he had a history of diabetes. Other endocrinological parameters suggested there is no other endocrinological impairment including insulinoma, prolactinoma, GH deficiency, or impaired hypothalamic-pituitary-adrenal, hypothalamic-pituitary-gonadal, and hypothalamic-pituitary-thyroid axes (basal laboratory data shown in Table 1).

**Table 1:** Laboratory data *All endocrinological data and some blood chemistry data were obtained at the morning resting position. ^#^Some data was obtained on different days because of the insufficient sample amount or conditions. HbA1c, hemoglobin A1c; CPR, C-peptide immunoreactivity; IRI, immunoreactive insulin; GAD, glutamic acid decarboxylase; HDL, high-density lipoprotein; LDL, low-density lipoprotein; AST, aspartate aminotransferase; ALT, alanine aminotransferase; LDH, lactate dehydrogenase; BUN, blood urea nitrogen; ACR, urinary albumin-to-creatinine ratio; ACTH, adrenocorticotropic hormone; GH, growth hormone; IGF-1, insulin-like growth factor-1; PRA, plasma renin activity; PAC, plasma aldosterone concentration; LH, luteinizing hormone; FSH, follicle-stimulating hormone; PRL, prolactin; TSH, thyroid-stimulating hormone; PTH, parathyroid hormone; WBC, white blood cell; RBC, red blood cell

Blood chemistry*				Endocrinological data*		
HbA1c (%)		7.3		ACTH (pg/mL)^#^		14.9
Blood glucose (mg/dL)		245		Cortisol (mg/dL)^#^		8.2
Anti-GAD antibody (U/mL)		<5.0		GH (ng/mL)		4.42
Anti-insulin antibody (U/mL)		3.6		IGF-1 (ng/mL)		364
Total cholesterol (mg/dL)		110		PRA (ng/mL/hr)		2.5
Triglyceride (mg/dL)		95		PAC (ng/dL)		16.5
HDL cholesterol (mg/dL)		37		LH (mIU/mL)		4.3
LDL cholesterol (mg/dL)		61		FSH (mIU/mL)		15.0
Total protein (g/dL)		6.7		PRL (ng/mL)		8.2
Albumin (g/dL)		3.0		TSH (mIU/mL)		1.221
Total bilirubin (mg/dL)		0.29		Free T3 (pg/mL)		2.13
AST (U/L)		8		Free T4 (ng/dL)		1.15
ALT (U/L)		10		Intact PTH (pg/mL)		44
LDH (U/L)		126				
Choline esterase (U/L)		235		Hematology		
BUN (mg/dL)		22.7		WBC (x10^3^/mL)		4.8
Creatinine (mg/dL)		0.75		RBC (x10^6^/mL)		3.54
Uric acid (mg/dL)		4.5		Hemoglobin (g/dL)		11.5
Creatine kinase (U/L)		40		Hematocrit (%)		33.7
Na (mEq/L)		137		Platelets (x10^3^/mL)		235
K (mEq/L)		4.4				
Cl (mEq/L)		105		Urine^#^		
Ca (mg/dL)		9.6		Glucose		(-)
IP (mg/dL)		3.9		Blood		(-)
Mg (mg/dL)		2.3		Ketones		(-)
				Protein		(-)
				ACR (mg/g・Cr )		36.4

**Figure 4:**
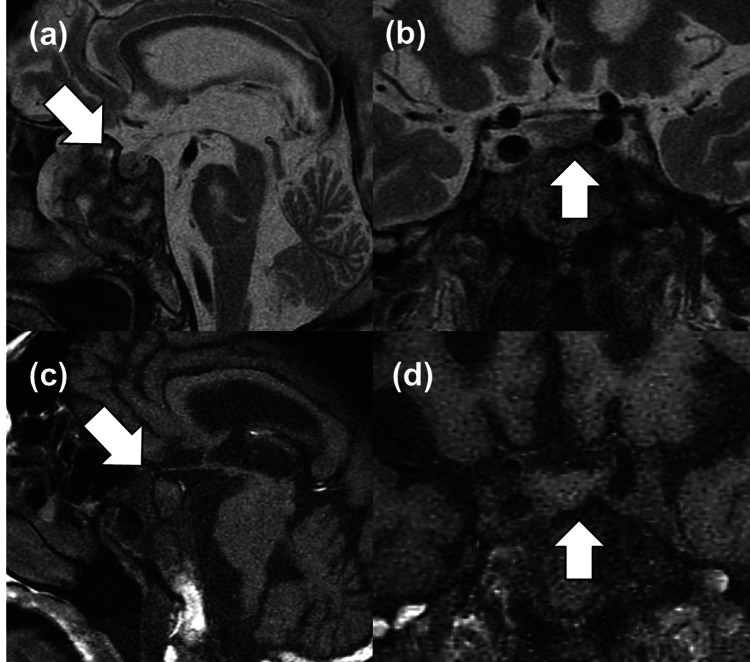
Images of the pituitary tumor Non-contrast coronal and sagittal T2- (a and b) and T1-weighted (c and d) pituitary images with MRI are shown. White arrows indicate a mass. MRI, magnetic resonance imaging

In the CT image, there is no mass or thinning of the adrenal gland suggesting adrenal insufficiency or pancreatic tumor. Consistently, a pituitary tumor (7x10 mm) was confirmed (Figure 4), and we diagnosed him with acromegaly.

Homonymous hemianopsia was not consistent with the tumor size as chiasm compression was intact. The thyrotropin-releasing hormone (TRH) test showed paradoxically increased GH, which peaked at 127.0 ng/mL at 30 minutes (Table 2).

**Table 2:** The result of the TSH test Abbreviations: GH, growth hormone; TSH, thyroid-stimulating hormone; PRL, prolactin

Minutes after stimulation	0	15	30	60	90	120
GH (ng/mL)	5.9	102.0	127.0	93.5	40.9	26.5
TSH (mIU/mL)	1.531	4.415	6.830	7.666	7.060	6.003
PRL (ng/mL)	10.1	51.8	45.5	37.0	27.4	21.1

**Figure 5:**
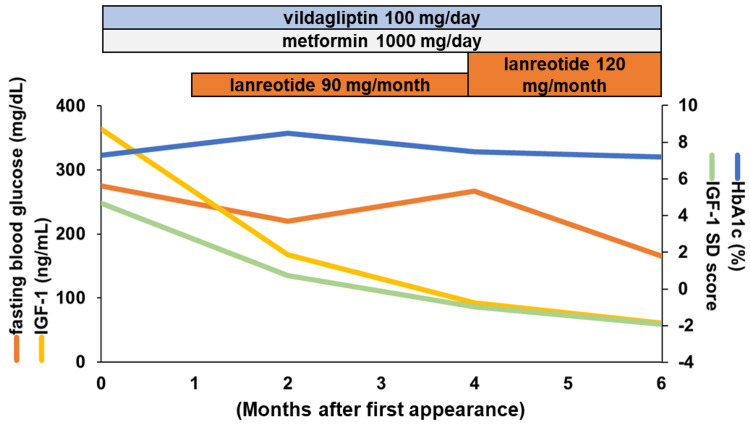
Treatment time-course of the present case Laboratory data and treatment time-course of the present case are shown. HbA1c, hemoglobin A1c; IGF-1, insulin-like growth factor-1; SD, standard deviation

Other acromegalic complications were also confirmed, including cauliflower-like tufting of the finger bones, heel pad thickness (27.0 mm), sleep apnea syndrome (apnea-hypopnea index; 19.2), and carpal tunnel syndrome. Otherwise, there were no other tumors in CT, including pancreatic tumors. Finally, he denied TSS, so we proposed the treatment of SSAs, lanreotide, which was started at 90 mg/month after the diagnosis of acromegaly. We restarted OHA immediately based on the increased blood glucose at first appearance. Considering anti-insulin antibodies and the risk of hypoglycemia, we did not use an insulin injection and restarted OHA with a decreased dosage. We additionally introduced nutritional guidance to take more dietary protein (i.e., 1.2-1.5 g/kg body weight/day) for sarcopenia. Four months after the first appearance, we were concerned that prolonged elevated glucose profiles (blood glucose: 267 mg/dL and HbA1c: 7.5%) might affect decreased IGF-1 levels (93 ng/mL) despite increased GH (6.64 ng/mL). However, an increase in OHA dosage could bring hypoglycemia. Therefore, we increased lanreotide to 120 mg, resulting in 166 mg/dL of blood glucose and HbA1c of 7.2% at six months that accompanied IGF-1 within +/-2.0 SDS without symptoms or hypoglycemia (Figure 5).

However, in the follow-up period, he complained of back pain that revealed multiple spinal compression fractures probably due to acromegalic osteoporosis, leading to difficulty in moving [15]. Finally, he was unable to visit outpatient and died at retirement home from prostration.

## Discussion

The present case was unique because of its process of diagnosis via investigation of hypoglycemia in an elderly patient. The causes of hypoglycemia were assumed to be attributed to several factors as follows: (1) severely progressed sarcopenia, (2) the presence of anti-insulin antibodies, and (3) intake of OHAs. First, sarcopenia is the condition of the loss of muscle mass and function, often shown in elderly patients [16]. Under sarcopenia, malnutrition and the reduction of muscle results in glucose storage disruption, which might result in decreased glucose storage and hypoglycemia in patients with diabetes, although the chronic imbalance of diabetes is also associated with weight loss in the present case [17,18]. In contrast, GH works as the counterregulatory hormone against hypoglycemia, such as glucagon and epinephrine, via increasing gluconeogenesis in peripheral organs and decreasing glucose uptake or consumption in peripheral tissues, especially during fasting [6]. Therefore, patients with acromegaly are exposed to high GH, which increases gluconeogenesis in the skeletal muscle and liver with lipolysis and could lead to decreased glucose storage in the body over a long time. The effect of excess GH on the muscle is still unclear. Some athletes abuse GH to improve their performance, believing in GH-derived anabolic mechanisms, but apparent and long-term GH excess could worsen exercise capacity and quality [19-21].

Additionally, older people tend to show sarcopenia, i.e., loss of muscle mass and functions [22]. Particularly, sarcopenia could lead to hypoglycemia in older people with diabetes via a reduction in muscle mass, resulting in glucose storage disruption [17,23]. Interestingly, elderly acromegaly patients are reported to show increased sarcopenia compared to non-acromegaly subjects, like the present case [22]. Therefore, sarcopenia was thought to be the leading cause of hypoglycemia in this case. Second, insulin autoimmune syndrome (IAS) leads to episodes of hypoglycemia with positive insulin autoantibodies and markedly elevated serum insulin [24]. Though classical IAS does not have a history of insulin usage, nonclassical IAS-associated hypoglycemia could happen in patients with a history of insulin usage [25]. In the present case, an anti-insulin antibody was positive, and the presence of possible nonclassical IAS is undeniable, but we could not prove elevated serum insulin levels and recurrence of hypoglycemia after a visit to our hospital. Also, his inappropriately elevated titer of anti-insulin antibody compared to the history of insulin usage could be associated with human leukocyte antigen, although we could not confirm [26]. Finally, some OHA also could lead to hypoglycemia. Sulfonylureas shows the highest prevalence of hypoglycemia among OHA. Metformin or dipeptidyl peptidase-4 inhibitor (DPP-4i) is not assumed to be a high-risk or minimal cause of hypoglycemia [27,28]. However, elderly people with sarcopenia are reported to be at a higher risk of hypoglycemia with OHAs [18,29]. Other medications in the present case are not plausible causes of hypoglycemia. Therefore, these associations might also affect hypoglycemia in this case.

The treatment of acromegaly in elderly patients sometimes has risk factors around surgery, such as narrow airways, heart failure, diabetes, or other factors [30]. Mainly, elderly acromegalic patients show impaired cognitive functions, mobility, and nutrition status in the geriatric population [31]. Otherwise, the progression of medical treatments, such as SSA, in acromegaly enables increased life expectancy, bringing endocrinologically well-controlled status without operation [3]. Accordingly, some elderly patients do not undergo TSS in clinical practice, although data on acromegaly in the elderly are still sparse [2,3]. In the present case, the low signal in T2-weighted MRI indicated densely-granulated type and significant response to the TRH test suggested good GH response to SSAs [4,32]. Lanreotide monotherapy showed normalized IGF-1 levels with improved glucose profiles without an increase of OHAs after SSA treatment. Correctively, geriatric multidimensional assessment is vital in elder patients with acromegaly based on their requests and expected outcomes.

Finally, during the coronavirus disease 2019 (COVID-19) pandemic, diagnostic and management dilemmas in endocrinological disease have emerged, especially picture diagnosis of acromegaly [33]. In summary, if the patients showed specific phenotypes suggesting acromegaly, such as progressed diabetic retina or physical findings, we should doubt the presence of acromegaly, even with hypoglycemia or uncontrolled and long-duration diabetes.

## Conclusions

To our knowledge, this is the first report of acromegaly diagnosed through hypoglycemia assessment. Advanced diabetic retinopathy works as the clue for diagnosing acromegaly, which represents a unique microvascular complication with diabetes, although the duration of diabetes could affect progressed diabetic retinopathy. GH is sometimes abused because of its anabolic effects to induce muscle hypertrophy. However, elderly patients with acromegaly are reported to encounter muscle weakness and sarcopenia. Thus, the impact of long-term excess GH is still unclear. Elderly people often show untypical phenotypes when developing a disease, which sometimes leads to delayed diagnosis. Therefore, clinicians should be careful when diagnosing elderly patients. In the future, with a super-aging society, acromegaly with untypical phenotypes could increase. We should conduct precise assessments in such cases.
